# Pressure-Mediated Biofeedback With Pelvic Floor Muscle Training for Urinary Incontinence: A Randomized Clinical Trial

**DOI:** 10.1001/jamanetworkopen.2024.42925

**Published:** 2024-11-05

**Authors:** Xiuqi Wang, Jin Qiu, Dan Li, Zhongmin Wang, Yanjing Yang, Guorong Fan, Xiaoyan Mao, Jiandi Wang, Shan Gao, Xihui Zhu, Tao Xu, Zhijing Sun

**Affiliations:** 1 Department of Obstetrics and Gynecology, Peking Union Medical College Hospital, Peking Union Medical College, Chinese Academy of Medical Sciences, National Clinical Research Center for Obstetric and Gynecologic Diseases, Beijing, China; 2 Department of Obstetrics and Gynecology, Tongren Hospital, Shanghai Jiao Tong University, Shanghai, China; 3 Department of Obstetrics and Gynecology, Shunyi Maternal and Children’s Hospital of Beijing, Beijing, China; 4 Department of Obstetrics and Gynecology, Dalian Women and Children’s Medical Center, Liaoning, China; 5 Department of Obstetrics and Gynecology, The Fourth Hospital of Shijiazhuang (Shijiazhuang Obstetrics and Gynecology Hospital), Heibei, China; 6 Department of Epidemiology and Biostatistics, Institute of Basic Medical Sciences, Chinese Academy of Medical Sciences and School of Basic Medicine, Peking Union Medical College, Beijing, China

## Abstract

**Importance:**

Supervised pelvic floor muscle training (PFMT) has been recommended as the first-line treatment for women with stress urinary incontinence (SUI), but more evidence on whether adjunctive methods would provide additional benefits is needed.

**Objective:**

To compare the efficacy of PFMT with or without a home-based pressure-mediated biofeedback (BF) device.

**Design, Setting, and Participants:**

This multicenter assessor-blinded randomized clinical trial was conducted in the obstetric clinics of 5 participating tertiary hospitals in China. Participants included eligible women with new-onset postpartum SUI who were enrolled from March 28, 2022, to October 13, 2023.

**Intervention:**

All participants received 3 months of supervised PFMT and were randomized to either the intervention (PFMT with a home-based pressure-mediated BF device) or the control group (home-based PFMT).

**Main Outcome and Measures:**

The primary outcome was the severity of urinary incontinence evaluated by the International Consultation on Incontinence Questionnaire–Urinary Incontinence Short Form after 3 months of supervised PFMT. The secondary outcomes included the cure and improvement rates, PFM strength, quality of life, self-efficacy, and adherence.

**Results:**

A total of 452 participants (median age, 34 [IQR, 31-36] years; median body mass index [calculated as the weight in kilograms divided by the height in square meters], 23.71 [IQR, 21.37-25.97]; median time since delivery, 50 [IQR, 43-61] days) were included in the analysis, with 223 in the intervention group and 229 in the control group. Compared with the control group, the intervention group achieved a significantly greater reduction in incontinence severity (median, 3.00 [IQR, 1.00-6.00] vs 2.00 [IQR, 0-4.00] points; *z* = −3.05; *P* = .002), significantly increased cure rate (45 of 223 [20.2%] vs 20 of 229 [8.7%]; *z* = 12.02; *P* = .001) and improvement (132 of 223 [59.2%] vs 102 of 229 [44.5%]; *z* = 9.71; *P* = .002), significantly greater pelvic floor muscle strength (median, 26.00 [IQR, 17.00-38.00] vs 21.00 [IQR, 13.50-33.50] cm H_2_O; *z* = −2.28; *P* = .02), and a significantly greater correlation between subjective and objective adherence (*r* = 0.825 vs *r* = 0.627).

**Conclusion and Relevance:**

In this randomized clinical trial, the efficacy of pressure-mediated BF combined with PFMT was superior to that of PFMT alone. These findings support the use of pressure-mediated BF devices for improving treatment outcomes for patients with SUI.

**Trial Registration:**

ClinicalTrials.gov Identifier: NCT05115864

## Key Points

**Question:**

What is the efficacy of traditional supervised pelvic floor muscle training (PFMT) with home-based pressure-mediated biofeedback (BF) as an adjuvant method for the treatment of women with postpartum stress urinary incontinence (SUI)?

**Findings:**

In this randomized clinical trial that included 452 participants with postpartum SUI, those in the intervention group receiving PFMT with the pressure-mediated BF showed better treatment outcomes compared with the control group after 3 months of treatment.

**Meaning:**

These findings suggest that pressure-mediated BF is an effective adjuvant to PFMT to improve postpartum SUI.

## Teaser

This randomized clinical trial assesses whether 3 months of pelvic floor muscle training with a home-based pressure-mediated biofeedback device is superior to training alone among postpartum women with stress urinary incontinence.

## Introduction

Stress urinary incontinence (SUI) is the most common disorder involving pelvic floor dysfunction, which refers to the involuntary leakage of urine due to increased abdominal pressure. SUI has negative impacts on physical and psychological health, quality of life, and socioeconomic burden.^1,2^ Epidemiological evidence^3^ indicates that the prevalence of SUI varies from 25% to 45% among adult women. Moreover, pregnancy and childbirth are widely considered major risk factors for SUI due to the weakness of and damage to pelvic floor structures.^4,5,6^ Based on the present evidence, most women experience urinary incontinence for the first time during pregnancy and the postpartum period, with the prevalence ranging from 26% to 63%.^7,8^

Supervised pelvic floor muscle training (PFMT) with a duration of at least 3 months is strongly recommended in the current guidelines as the first-line treatment for SUI or stress-predominant urinary incontinence in women.^9,10,11^ Based on this recommendation, physicians have begun to wonder whether there is an adjunctive treatment method that could provide additional benefits over PFMT alone for women with SUI. To date, none of these methods have gained widespread acceptance, including biofeedback (BF), electrical stimulation, bladder diaries, and combined home and clinic programs.

However, the efficacy of pressure-mediated BF as an adjuvant method with traditional supervised PFMT for the treatment of postpartum SUI remains to be explored. The aim of our study was to determine whether PFMT with a home-based pressure-mediated BF device is superior to PFMT alone by evaluating SUI severity, rates of cure and improvement, PFM strength, quality of life, self-efficacy, and adherence among postpartum women after 3 months of supervised PFMT.

## Methods

### Study Design and Ethical Approval

The present study was designed as a multicenter assessor-blinded parallel-group randomized clinical trial (RCT) to evaluate the efficacy of PFMT with a home-based pressure-mediated BF device for postpartum women with new-onset SUI. The study design has been published previously,^12^ and the trial protocol is provided in Supplement 1. The study was approved by the ethics committee of Peking Union Medical College Hospital and followed the Consolidated Standards of Reporting Trials (CONSORT) reporting guidelines. Written informed consent was obtained from all participants before data collection.

### Participants

Participants who underwent regular postpartum checks from March 28, 2022, to October 13, 2023, were recruited from the obstetric clinics of 5 participating tertiary hospitals based on the inclusion and exclusion criteria. Women 18 years or older who were clinically diagnosed with new-onset SUI or stress-predominant urinary incontinence within 12 weeks after delivery following the definition recognized by international guidelines^1^ were eligible to participate. The exclusion criteria consisted of (1) urgency urinary incontinence alone, (2) third- and fourth-degree perineal tears, (3) diastasis recti abdominis and chronic pelvic pain, (4) prepregnancy SUI, (5) previous pelvic surgery, (6) malignant pelvic cancer, (7) urogenital infections, (8) receipt of formal PFMT in the past 5 years, (9) psychiatric or neurological disorders that interfered with cooperation with treatment procedures or the PFM contraction, and (10) inability to contract the PFMs.

### Randomization and Masking

Eligible participants were randomized to either the intervention group (PFMT with a home-based pressure-mediated BF device) or the control group (PFMT at home) with an allocation ratio of 1:1. Randomization was performed automatically via a computer-generated randomization program by entering the patient’s name and a copy of the consent form. Owing to the study design, the group allocation was revealed to the participants after randomization. The researchers who were responsible for recruitment, intervention delivery, and supervision (J.Q., D.L., Z.W., and Y.Y.) were not blinded to group allocation. However, the researchers who conducted the PFM assessments and data analysis (X.W., G.F., X.M., J.W., S.G., X.Z., and T.X.) were blinded to the allocation status.

### Intervention

After randomization, participants in both groups were assisted in installing the PFMT study app. At baseline, electronic questionnaires were completed by all participants via the app to collect demographic information and outcome measurements, and PFM assessments were performed by the same assessor (G.F., X.M., J.W., S.G., and X.Z.) at each participating center. Moreover, participants in the intervention group were given a demonstration video and a booklet created by the research physician, with explanations of the connection, operation, and maintenance of the home-based BF device. The participants were asked to report any difficulties they experienced while using the BF device. During the 3-month treatment period, both groups were given the PFMT program following the same training protocol, which contained 3 sets each day (each set lasted for 6 minutes). The PFMT sessions of both groups were supervised by a researcher (J.Q., D.L., Z.W., and Y.Y.) who telephoned the patients once every 3 weeks, and the app sent daily reminders to perform PFMT. Both groups were asked to complete the daily training via the app. The control group completed daily PFMT (without the BF device) following the video provided by the app. The intervention group performed daily PFMT with a personal home-based BF device (XFT-0010CK; Shenzhen XFT Medical Limited) following the video provided by the app. The device with an intravaginal insert was able to provide real-time visual BF on the progression of PFM contraction via the pressure collected by the vaginal air-filled probe during PFMT. The BF status was displayed on the screen of the app via short-range wireless technology (Bluetooth Special Interest Group). After 3 months of treatment, both groups were asked to complete the same questionnaires and PFM assessments again.

### Outcome Measures

#### Primary Outcome Measure

The primary outcome was the severity of urinary incontinence evaluated by a validated Chinese version of the International Consultation on Incontinence Questionnaire–Urinary Incontinence Short Form (ICIQ-UI SF).^13^ The ICIQ-UI SF score ranges from 0 to 21 points and can be divided into scores indicating mild (<5), moderate (5-13), and severe (>13) urinary incontinence for 3 sections (frequency, amount, and impact of urine leakage on life).^14,15,16^

#### Cure and Improvement of SUI

Cured SUI was defined as the total score on the ICIQ-UI SF of 0 at the end of 3 months of treatment. Improvement in SUI was defined as a reduction of 3 points or more in the ICIQ-UI SF score at the end of 3 months of treatment compared with baseline.^17^

#### PFM Strength

Subjective PFM strength was assessed via vaginal palpation and classified within the range of 0 to 5 on the modified Oxford grading scale, with higher grades reflecting better strength.^18^ Objective PFM strength was assessed via an intravaginal manometer and is presented as the maximum voluntary contraction pressure recorded in centimeters of water with a resolution of 0.01 cm H_2_O.^19^

#### Quality of Life

Quality of life was evaluated via the Chinese version of the Incontinence Quality of Life Instrument,^20^ with 22 items comprising 3 sections: (1) avoidance and limiting behavior; (2) psychosocial impacts; and (3) social embarrassment. The total score ranges from 0 to 100, with higher scores representing better quality of life.

#### Self-Efficacy

Self-efficacy was evaluated by the Broome Pelvic Muscle Self-Efficacy Scale.^21,22^ The total score ranges from 0 to 100; higher scores indicate greater self-efficacy perceived by the participant.

#### Completion and Adherence

In both groups, the daily completion of training was recorded by the training diary and PFMT video, whereas in the intervention group, it was also recorded by the intravaginal BF device. Subjective completion was defined as the training diary record, which was completed by participants via the PFMT study app, and objective completion was defined as the actual running time of the PFMT video recorded by the PFMT study app in both groups. Overall patient adherence was defined as the rate of completion and expectation during 3 months of supervised treatment and categorized as follows: less than 50% indicated low adherence; 50% to 75%, medium adherence; and 75% or more, high adherence.

### Statistical Analysis

The data were analyzed via SPSS, version 26.0 (IBM Corporation). In our study, most of the data were nonnormally distributed and are presented as medians (IQRs). The Wilcoxon signed rank test was used for analyses within groups, and the Mann-Whitney test was used for comparisons between groups. Categorical variables are presented as number (percentage), with the χ^2^ test used for within-group comparisons and the Fisher exact test used for between-group comparisons. The correlation between self-reported adherence and video-recorded adherence was assessed via Pearson analysis. Two-sided *P* < .05 indicated statistical significance.

The primary outcome of this study was the ICIQ-UI SF score. The sample size calculation was based on previous studies that detected a significant difference of 2.5 points in the ICIQ-UI SF score and an SD of 8.1 as the primary outcome between the groups.^23^ Based on this definition, a sample size of 222 participants per group was calculated via PASS15 software, version 15.0.5 (NCSS), with a power of 0.9 and a 2-sided significance level of .05 (α = .05, 1 − β = 0.9). Thus, a total of 500 women were recruited (250 women per group), allowing for approximately 10% loss at the end of the 3-month treatment.

## Results

### Baseline Demographic Characteristics of the Participants in Both Groups

**Figure 1.**
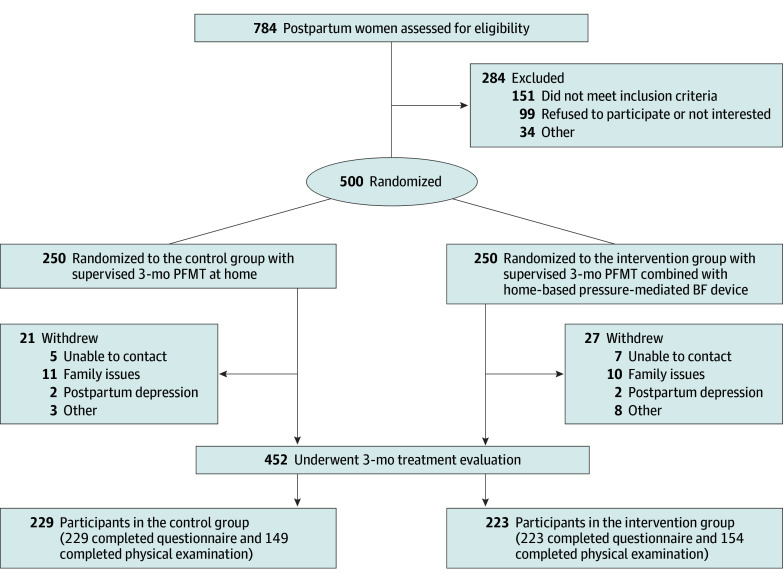
Flow Diagram of the Study BF indicates biofeedback; PFMT, pelvic floor muscle training.

The analytic sample included 452 participants (median age, 34 [IQR, 31-36] years; median prepregnancy body mass index [calculated as the weight in kilograms divided by the height in meters squared], 23.71 [IQR, 21.37-25.97]; median time since delivery, 50 [IQR, 43-61] days), with 223 participants in the intervention group and 229 in the control group (Table 1 and Figure 1). In the analysis of baseline demographic characteristics, there were no significant differences between the intervention and control groups in terms of age, body mass index, smoking status, gravidity and parity, delivery mode, newborn weight, prolonged second stage of labor, or the use of epidural anesthesia.

**Table 1.** Demographic Characteristics of Participants at Baseline

Characteristic	Participant group		
	All (N = 452)	Control (n = 229)	Intervention (n = 223)
Age, median (IQR), y	34 (31-36)	34 (31-36)	34 (31-36)
Height, median (IQR), cm	163 (160-166)	163 (160-167)	163 (160-166)
Prepregnancy weight, median (IQR), kg	56.20 (50.60-63.29)	55.50 (50.90-62.24)	56.96 (50.35-64.00)
Preintervention weight, median (IQR), kg	62.63 (56.00-70.00)	62.00 (56.50-69.00)	63.00 (56.00-70.00)
Preintervention BMI median (IQR),	21.30 (19.02-23.69)	20.84 (19.02-23.37)	21.63 (19.02-23.85)
Prepregnancy BMI median (IQR),	23.71 (21.37-25.97)	23.44 (21.32-25.88)	24.14 (21.48-26.17)
Weight gain during pregnancy, kg	13.00 (10.00-17.00)	13.00 (10.00-17.25)	13.00 (10.00-17.00)
University education, No. (%)			
Yes	409 (90.5)	204 (89.1)	205 (91.9)
No	43 (9.5)	25 (10.9)	18 (8.1)
Time after delivery, median (IQR), d	50 (43-61)	49 (43-60)	52 (44-63)
Menses, No. (%)			
Yes	48 (10.6)	21 (9.2)	27 (12.1)
No	404 (89.4)	208 (90.8)	196 (87.9)
Feeding patterns, No. (%)			
Breastfeeding	240 (53.1)	116 (50.7)	124 (55.6)
Artificial feeding	25 (5.5)	13 (5.7)	12 (5.4)
Both	187 (41.4)	100 (43.7)	87 (39.0)
Occupation position, No. (%)			
Standing mainly	52 (11.5)	27 (11.8)	25 (11.2)
Sitting mainly	368 (81.4)	184 (80.3)	184 (82.5)
Others	32 (7.1)	18 (7.9)	14 (6.3)
Occupation mode, No. (%)			
Mental mainly	336 (74.3)	166 (72.5)	170 (76.2)
Manual mainly	17 (3.8)	8 (3.5)	9 (4.0)
Both	99 (21.9)	55 (24.0)	44 (19.7)
Physical labor, No. (%)			
Light	290 (64.2)	148 (64.6)	142 (63.7)
Moderate	158 (35.0)	81 (35.4)	77 (34.5)
Heavy	4 (0.9)	0	4 (1.8)
Smoking, No. (%)			
Yes	7 (1.5)	3 (1.3)	4 (1.8)
No	445 (98.5)	226 (98.7)	219 (98.2)
Constipation, No. (%)			
Yes	147 (32.5)	74 (32.3)	73 (32.7)
No	305 (67.5)	155 (67.7)	150 (67.3)
Asthma, No. (%)			
Yes	91 (20.1)	48 (21.0)	43 (19.3)
No	361 (79.9)	181 (79.0)	180 (80.7)
Family history of urinary incontinence, No. (%)			
Yes	103 (22.8)	54 (23.6)	49 (22.0)
No	349 (77.2)	175 (76.4)	174 (78.0)
Family history of pelvic organ prolapse, No. (%)			
Yes	26 (5.8)	12 (5.2)	14 (6.3)
No	426 (94.2)	217 (94.8)	209 (93.7)
No. of births, median (IQR)			
Cesarean section	0 (0-0)	0 (0-0)	0 (0-0)
Vaginal delivery	1.00 (1.00-1.75)	1.00 (1.00-2.00)	1.00 (1.00-1.00)
Abortion history, median (IQR) No.	0 (0-0)	0 (0-0)	0 (0-0)
Mode of delivery, No. (%)			
Vaginal delivery	400 (88.5)	200 (87.3)	200 (89.7)
Cesarean section	52 (11.5)	29 (12.7)	23 (10.3)
Vaginal delivery, No. (%)^a^			
Episiotomy	143 (35.8)	71 (35.5)	72 (36.0)
Perineal laceration	273 (68.3)	136 (68.0)	137 (68.5)
Forceps birth	19 (4.8)	10 (5.0)	9 (4.5)
Epidural anesthesia	269 (67.3)	140 (70.0)	129 (64.5)
Newborn weight, median (IQR), g	3350.00 (3050.00-3637.50)	3350.00 (3050.00-3615.00)	3340.00 (3050.00-3650.00)
Precipitate labor, No. (%)^a^			
Yes	57 (14.2)	27 (13.5)	30 (15.0)
No	343 (85.8)	173 (86.5)	170 (85.0)
Prolonged second stage, No. (%)^a^			
Yes	57 (14.3)	31 (15.5)	26 (13.0)
No	343 (85.8)	169 (84.5)	174 (87.0)

Abbreviation: BMI, body mass index (calculated as the weight in kilograms divided by the height in square meters).

^a^ Includes 400 women with vaginal deliveries.

### Primary Outcome

The ICIQ-UI SF total score was not significantly different between the groups at baseline; however, the median difference in the ICIQ-UI SF score from baseline to the end of the intervention was significantly greater in the intervention group than in the control group (3.00 [IQR, 1.00-6.00] vs 2.00 [IQR, 0-4.00] points; *z* = −3.05; *P* = .002), including the frequency (1.00 [IQR, 0-1.00] vs 0 [0-1.00]; *z* = −2.96; *P* = .003), amount (0 [IQR, 0-2.00] vs 0 [IQR, 0-0]; *z* = −2.40; *P* = .02), and impact of urine leakage on life (2.00 [IQR, 0-3.00] vs 1.00 [IQR, 0-3.00]; *z* = −2.05; *P* = .04) (Table 2). Compared with those in the control group, the intervention group showed significant improvement after 3 months of treatment among participants with mild SUI (median, 3.00 [IQR, 0.25-4.00] vs 4.00 [IQR, 3.00-4.00] points; *z* = −2.67; *P* = .008) and among those with moderate SUI (median, 4.00 [IQR, 3.00-5.75] vs 5.00 [IQR, 4.00-7.00] points; *z* = −2.09; *P* = .04), whereas improvement in severe SUI did not differ significantly between the groups.

**Table 2.** Primary Outcome of ICIQ-UI SF at Baseline and at 3 Months of Treatment^a^

Outcome measure	Participant group		χ^2^ test *z* score	*P* value
	Control (n = 229)	Intervention (n = 223)		
**ICIQ-UI SF total score**				
Baseline	6.00 (4.00-9.00)	7.00 (4.00-9.00)	−0.72	.47
3-mo	4.00 (3.00-6.00)	4.00 (2.00-5.00)	−3.03	.002
*z* Score	−9.14	−10.90	NA	NA
*P* value within group	<.001	<.001	NA	NA
Difference	2.00 (0-4.00)	3.00 (1.00-6.00)	−3.05	.002
**Severity of SUI**				
Mild				
Baseline	4.00 (3.00-5.00)	4.00 (3.00-5.00)	−1.04	.30
3-mo	4.00 (3.00-4.00)	3.00 (0.25-4.00)	−2.67	.008
*z* Score	−2.21	−4.87	NA	NA
*P* value within group	.03	<.001	NA	NA
Moderate				
Baseline	8.00 (7.00-9.00)	8.00 (7.00-9.00)	−0.04	.97
3-mo	5.00 (4.00-7.00)	4.00 (3.00-5.75)	−2.09	.04
*z* Score	−7.91	−8.56	NA	NA
*P* value within group	<.001	<.001	NA	NA
Severe				
Baseline	15.00 (13.75-16.00)	15.00 (13.00-16.00)	−0.67	.50
3-mo	6.50 (4.00-9.00)	6.00 (3.00-8.00)	−1.21	.23
*z* Score	−4.07	−4.15	NA	NA
*P* value within group	<.001	<.001	NA	NA
**Frequency of urine leakage**				
Baseline	1.00 (1.00-2.00)	1.00 (1.00-2.00)	−0.59	.56
3-mo	1.00 (1.00-1.00)	1.00 (1.00-1.00)	−2.83	.005
*z* Score	−7.93	−9.74	NA	NA
*P* value within group	<.001	<.001	NA	NA
Difference	0 (0-1.00)	1.00 (0-1.00)	−2.96	.003
**Amount of urine leakage**				
Baseline	2.00 (2.00-2.00)	2.00 (2.00-2.00)	−0.06	.96
3-mo	2.00 (2.00-2.00)	2.00 (1.00-2.00)	−2.80	.005
*z* Score	−6.50	−7.78	NA	NA
*P* value within group	<.001	<.001	NA	NA
Difference	0 (0-0)	0 (0-2.00)	−2.40	.02
**Impact on life**				
Baseline	3.00 (1.00-5.00)	3.00 (1.00-5.00)	−0.59	.56
3-mo	1.00 (0-2.00)	1.00 (0-2.00)	−2.95	.003
*z* Score	−7.53	−9.52	NA	NA
*P* value within group	<.001	<.001	NA	NA
Difference	1.00 (0-3.00)	2.00 (0-3.00)	−2.05	.04

Abbreviations: ICIQ-SF UI, International Consultation on Incontinence Questionnaire-Urinary Incontinence Short Form; NA, not applicable; SUI, stress urinary incontinence.

^a^ Scores are reported as median (IQR) and range from 0 to 21 points and can be divided into scores indicating mild (<5), moderate (5-13), and severe (>13) urinary incontinence for 3 sections (frequency, amount, and impact of urine leakage on life).^14,15,16^

### Secondary Outcomes

#### Outcomes at Follow-Up and PFM Strength

Outcomes at follow-up were based on the ICIQ-UI SF score. More participants in the intervention group than in the control group were considered to have achieved a cure (45 of 223 [20.2%] vs 20 of 229 [8.7%]; *z* = 12.02; *P* = .001) or improvement (132 of 223 [59.2%] vs 102 of 229 [44.5%]; *z* = 9.71; *P* = .002) in SUI at the end of the intervention period (Table 3). The maximum voluntary contraction pressure of the intervention group was significantly greater than that of the control group at the end of the 3-month treatment (median, 26.00 [IQR, 17.00-38.00] vs 21.00 [IQR, 13.50-33.50] cm H_2_O; *z* = −2.28; *P* = .02) (Table 3).

**Table 3.** Secondary Outcomes at Baseline and 3 Months of Treatment

Outcome measure	Participant group		χ^2^ test *z* score	*P* value
	Control (n = 229)	Intervention (n = 223)		
**Cure or improvement, No. (%)**				
Cure	20 (8.7)	45 (20.2)	12.02	.001
Improvement	102 (44.5)	132 (59.2)	9.71	.002
**PFM strength**				
Subjective PFM strength on MOS, No. (%)^a^				
Baseline				
≤3	137 (91.9)	137 (89.5)	−0.72	.47
>3	12 (8.1)	16 (10.5)		
3-mo				
≤3	90 (60.4)	81 (52.9)	−1.31	.19
>3	59 (39.6)	72 (47.1)		
*z* Score	−6.86	−7.00	NA	NA
*P* value within group	<.001	<.001	NA	NA
Objective PFM strength, cm H_2_O				
VRP, median (IQR)				
Baseline	45.00 (26.50-65.00)	46.00 (22.00-68.50)	−0.28	.78
3-mo	49.00 (31.50-68.00)	48.00 (33.00-68.50)	−0.19	.85
*z* Score	−3.70	−2.69	NA	NA
*P* value within group	<.001	.007	NA	NA
MVCP, median (IQR)				
Baseline	16.00 (9.00-23.50)	19.00 (11.00-29.50)	−1.82	.07
3-mo	21.00 (13.50-33.50)	26.00 (17.00-38.00)	−2.28	.02
*z* Score	−6.29	−5.93	NA	NA
*P* value within group	<.001	<.001	NA	NA
**I-QOL** ^b^				
Avoidance and limiting behavior, median (IQR)				
Baseline	32.00 (27.00-36.00)	32.00 (27.00-36.00)	−0.25	.80
3-mo	34.00 (30.00-38.00)	35.00 (32.00-38.00)	−0.64	.52
*z* Score	−5.71	−6.83	NA	NA
*P* value within group	<.001	<.001	NA	NA
Psychosocial impacts, median (IQR)				
Baseline	40.00 (34.00-44.00)	38.00 (33.00-44.00)	−0.84	.40
3-mo	43.00 (39.00-45.00)	43.00 (38.00-45.00)	−0.76	.45
*z* Score	−7.17	−7.61	NA	NA
*P* value within group	<.001	<.001	NA	NA
Social embarrassment, median (IQR)				
Baseline	19.00 (15.00-22.00)	19.00 (16.00-23.00)	−0.14	.89
3-mo	21.00 (20.00-25.00)	21.00 (20.00-25.00)	−0.37	.72
*z* Score	−8.94	−9.76	NA	NA
*P* value within group	<.001	<.001	NA	NA
I-QOL total scores, median (IQR)				
Baseline	91.00 (76.00-101.00)	89.00 (76.00-102.00)	−0.42	.68
3-mo	98.00 (89.00-107.00)	98.00 (88.00-107.00)	−0.05	.96
*z* Score	−8.20	−8.74	NA	NA
*P* value within group	<.001	<.001	NA	NA
BPMSES total scores, median (IQR)^c^				
Baseline	81.00 (66.50-93.00)	81.00 (68.00-93.00)	−0.28	.78
3-mo	72.00 (60.00-85.00)	78.00 (62.00-91.00)	−2.46	.02
*z* Score	−6.90	−4.39	NA	NA
*P* value within group	<.001	<.001	NA	NA
Completion and adherence, median (IQR)				
Subjective completion, min	14.00 (12.00-15.00)	14.00 (12.00-15.00)	−0.46	.65
Objective completion, min	13.00 (10.00-15.00)	13.00 (10.25-14.00)	−1.13	.26
Use time of the intravaginal insert, min	NA	14.00 (11.00-15.00)	NA	NA
Subjective adherence, No. (%)^d^				
>75%	96 (43.0)	94 (43.5)	0.59	.75
50%-75%	88 (39.5)	79 (36.6)		
<50%	39 (17.5)	43 (19.9)		
Objective adherence, No. (%)^d^				
>75%	154 (69.1)	151 (69.9)	2.34	.31
50%-75%	45 (20.2)	34 (15.7)		
<50%	24 (10.8)	31 (14.4)		
Seek other treatment, No. (%)	6 (2.7)	7 (3.1)	0.11	.74

Abbreviations: BPMSES, Broome Pelvic Muscle Self-Efficacy Scale; I-QOL, Incontinence Quality of Life Instrument; MOS, modified Oxford grading scale; MVCP, maximum voluntary contraction pressure; PFM, pelvic floor muscle; VRP, vaginal resting pressure.

^a^ Classifications range from 0 to 5, with higher grades reflecting better strength.

^b^ The I-QOL contains 22 items and each ranging from 1 to 5 points. Subscale scores range from 8 to 40 points in avoidance and limiting behavior, 9 to 45 points in the section of psychosocial impacts, and 5 to 25 points in the section of social embarrassment, and total scores range from 0 to 100, with higher scores representing better quality of life.

^c^ Scores range from 0 to 100; higher scores indicate greater self-efficacy perceived by the participant.

^d^ Due to lack of identification after data storage and export, there were 223 of 229 cases in the control group and 216 of 223 cases in the intervention group available for the final analysis of subjective and objective adherence.

#### Quality of Life and Self-Efficacy

Compared with baseline, quality of life after 3 months of treatment improved significantly in the control group (median, 91.00 [IQR, 76.00-101.00] vs 98.00 [IQR, 89.00-107.00] points; *z* = −8.20; *P* < .001) and in the intervention group (median, 89.00 [IQR, 76.00-102.00] vs 98.00 [IQR, 88.00-107.00] points; *z* = −8.74; *P* < .001), but there was no significant difference between the groups in any of the 3 domains (Table 3). For self-efficacy, no significant difference between the groups was found at baseline. At 3 months, the control group had significantly lower Broome Pelvic Muscle Self-Efficacy Scale scores than the intervention group (median, 72.00 [IQR, 60.00-85.00] vs 78.00 [IQR, 62.00-91.00] points; *z* = −2.46; *P* = .02).

#### Completion and Adherence

**Figure 2.**
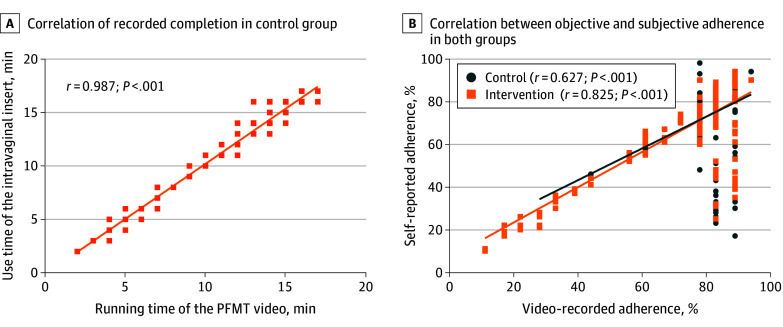
Scatterplot of Correlation of Completion and Adherence in the Control and Intervention Groups A, The orange line shows the correlation between the completion recorded by the running time of the pelvic floor muscle training (PFMT) video and the use time of the intravaginal insert in the intervention group. B, The blue line marks the correlation between subjective adherence (self-reported) and objective adherence (video-recorded) in the control group. The orange line marks the correlation between subjective adherence (self-reported) and objective adherence (video recorded) in the intervention group. Blue dots represent the subjective and objective adherence in the control group; orange boxes, subjective and objective adherence in the intervention group

No difference in subjective or objective completion was detected between the groups (Table 3). More participants were considered to have high adherence objectively in the intervention group than in the control group (151 of 216 [69.9%] vs 154 of 223 [69.1%]). The correlation between the use time of the intravaginal insert and the running time of the PFMT video in the intervention group was highly positive (*r* = 0.987; *P* < .001) (Figure 2A). The correlation between subjective and objective adherence was greater in the intervention group than in the control group (*r* = 0.825 vs *r* = 0.627) (Figure 2B).

### Adverse Events and Difficulties

Among all the participants in the intervention group, no device-related serious adverse events were reported. Only 4 participants (1.8%) reported pain, and 1 (0.4%) reported bacterial vaginosis. Fifteen participants (6.7%) reported various difficulties, categorized as before, during, and after the use of the device. Two participants (0.9%) reported difficulties connecting via wireless technology or fitting the insert into the vagina. Twelve participants (5.4%) reported difficulties regarding the movement of the intravaginal insert (out and/or deep) and undefined discomfort. One participant (0.4%) reported cleaning difficulty.

## Discussion

This RCT revealed that pressure-mediated BF with PFMT was superior to PFMT alone in improving SUI severity, rates of cure and improvement, and PFM strength. The correlation between subjective and objective adherence was greater in the intervention group than in the control group. These findings provide more evidence for clinicians to consider pressure-mediated BF as an adjuvant method to PFMT for women with postpartum urinary incontinence who are seeking better PFMT treatment outcomes and to reduce pressure on the health care system.

However, whether pressure-mediated BF could provide additional benefits in the treatment of SUI is still under debate. Some guidelines recommend PFMT with the addition of BF for women with SUI, especially those who are unable to actively contract their PFMs or for whom PFMT alone is not sufficiently effective.^24,25^ According to published studies, the different types include motion-based, electromyographic-mediated, pressure-mediated, and sensation-mediated BF.^26,27,28,29,30,31,32^ The BF device used in our study involves pressure-mediated recordings of squeeze pressure during a voluntary PFM contraction, and the presentation of this information to the woman is visually depicted in the app in graphical form. On the interface of the app, 2 curves are shown: one is depicted according to the real-time pressure signal to show real-time PFM contractions, and the other is provided in advance to guide the participants during PFMT. As a result, the participants were not only aware of the status of PFM activity but also accurately recognized improper performance and correct PFM contraction in real time during PFMT. This might be one of the reasons why PFMT with the pressure BF device was more effective than PFMT alone in our study.

Previous studies on the use of pressure-mediated BF in postpartum women are scarce. However, a few studies conducted on different BF devices or populations reported similar results, indicating that PFMT with BF could be more beneficial than PFMT alone.^33,34,35,36,37,38^ An RCT of 72 participants^33^ revealed that the addition of outpatient pressure-mediated BF to home-based PFMT for SUI management resulted in a superior objective cure but did not increase the frequency of home exercise sets, PFM strength, or quality of life. In contrast to the patients in our study who completed the pressure-mediated BF training at home daily, the patients in the previous study completed the pressure-mediated BF training following an outpatient protocol twice a week. Another RCT using a motion-based device to guide PFMT at home compared with a home-based PFMT^34,35^ reported superior improvement of urinary incontinence symptoms after 2 years.

However, whether the efficacy of PFMT with BF is superior to that of PFMT alone has still not been confirmed because some other studies reported results that are inconsistent with our findings.^39,40,41,42^ An RCT of 32 participants^40^ compared PFMT alone, PFMT with pressure-mediated BF, and PFMT with electrical stimulation for the home treatment of urinary incontinence and demonstrated no difference in improvement in quality of life, PFM strength, or urine leakage episodes between groups. Two other studies (with 21 and 45 participants)^41,42^ reported that supervised electromyographic BF–assisted PFMT did not improve sexual function, SUI symptoms, or quality of life. The inconsistent conclusions of these published studies are likely related to low methodological quality, heterogeneity of the outcomes, and differences in the implementation of the intervention protocols and BF modalities.

Based on these studies, a significant reduction in PFM strength has been reported among postpartum women, and in another study,^43^ this decrease was shown to be a modifiable risk factor for the later occurrence and development of pelvic floor dysfunction. In our study, significantly greater PFM strength was detected in the intervention group than in the control group. This finding is in accordance with another study^44^ in which PFM strength was significantly greater in women with SUI in the intravaginal pressure-mediated BF group than in the group receiving PFMT alone. These findings indicate that changes in PFM strength may reflect clinical symptoms to some extent.

Additionally, in our study, the participants in the intervention group with mild and moderate SUI at baseline showed significant improvement after 3 months of treatment compared with those in the control group, whereas severe SUI did not differ significantly between the groups. This finding is in accord with another study, which may explain our findings,^45^ that evaluated the efficacy of BF and electrostimulation-assisted PFMT for women with mild and moderate to severe SUI. This study revealed significant improvements in SUI symptoms and PFM strength in the group with mild SUI but not in the group with moderate to severe SUI.^45^ This difference is considered related to more severe fibrosis and muscle fiber atrophy with increased disease severity, which limits significant improvement in women with severe SUI.^45^ It is suggested that if conservative treatment is inadequate for patients with more severe disease, it is vital to notify them of the restrictions and recommend that they undergo surgery to decrease health care expenses.

### Limitations

This study has some limitations. The participants were not blinded to treatment assignment. To improve the study design in the future, we will add comorbid illnesses (eg, hypertension and diabetes) to the data collected at baseline, and we will adopt surface electromyography based on the Glazer protocol to assess PFM strength and endurance.^46^ The results of the 3-month intervention have been reported, but the long-term effectiveness of the use of pressure-mediated BF devices for the treatment of postpartum SUI needs to be explored and reported in the future.

## Conclusions

This RCT found that the efficacy of a 3-month supervised home-based pressure-mediated BF training for postpartum SUI was superior to that of PFMT alone, with a greater correlation between self-reported and video-recorded adherence. These findings support the idea that pressure-mediated BF devices enable clinicians to precisely monitor patients and reduce pressure on the health care system. More importantly, this study offers more evidence for considering pressure-mediated BF as an adjuvant method to PFMT to improve treatment outcomes for patients with SUI.

## Notes

Supplement 1.
Trial Protocol

Supplement 2.
Data Sharing Statement
