# Supplementary material for: Pressure-Mediated Biofeedback With Pelvic Floor Muscle Training for Urinary Incontinence: A Randomized Clinical Trial
Source: JAMA Netw Open. 2024 Nov 5;7(11):e2442925. doi: 10.1001/jamanetworkopen.2024.42925 (PMC11539013; doi:10.1001/jamanetworkopen.2024.42925)
Supplement: Supplement 1. — Trial Protocol [file jamanetwopen-e2442925-s001.pdf]

TRIAL PROTOCOL

Introduction

Supervised pelvic floor muscle training (PFMT) of at least 3 months duration has been strongly recommended as a first-line treatment for women with stress urinary incontinence (SUI) or SUI-predominant mixed urinary incontinence (MUI), including elderly and postnatal women. However, for the treatment of SUI and MUI in postpartum women, it is currently uncertain whether supervised PFMT combined with a biofeedback device is superior to PFMT alone. Despite some supportive results, more reliable evidence is lacking.

Study design

The study is designed as a multicentre assessor-blinded parallel-group randomized controlled trial, comparing PFMT with a home-based pressure-mediated biofeedback device (intervention group) and PFMT at home (control group) for women with new-onset SUI or SUI-predominant MUI after delivery. Patient recruitment has been conducted in 5 tertiary hospitals since March 2022 and is expected to last for up to 24 months.

Patient selection and recruitment

Since March 2022, women who present for regular postpartum clinic visits at the 5 participating tertiary hospitals and report new-onset SUI or SUI-predominant MUI within three months after delivery will be instructed on the project. Afterwards, the well-trained physicians in each participating centre will screen the potentially eligible patients. Patients who meet all the criteria and express an interest in participating will be recruited. After participant eligibility is confirmed and consent obtained, the site physicians will complete the randomization by

entering the patient’s name, date of birth, phone number and picture of the consent form into the computer-generated randomization program in real time. The allocation status will not be concealed from nonblinded physicians who will perform the recruitment, supervision and follow-up or from the patients but will be concealed from the assessment physicians. The inclusion and exclusion criteria are as follows:

Inclusion Criteria: 1) 6 weeks＜after delivery<3 months postpartum; 2) Clinically diagnosed SUI as the primary problem. We will consider SUI and MUI following the definition recognized by international guidelines; 3) Women aged 18 years or older

Exclusion Criteria: 1) Urgency UI alone; 2) Pelvic Organ Prolapse Quantification (POP-Q) stage ≥2; 3) Third- and fourth-degree perineal tears; 4) Presence of diastasis recti abdominis and chronic pelvic pain as the primary problem needing treatment; 5) A history of SUI before pregnancy; 6) Previous pelvic surgery; 7) Malignant pelvic cancer; 8) Urogenital infections; 9) Receipt of formal instruction on PFMT in the past 5 years; 10) Unsuitability to participate because of significant diseases; 11) Others: Inability to contract the PFMs on digital examination when requested; inability to use the device in the vagina.

Randomization and masking

After evaluation of the inclusion criteria and assessment at baseline, patients will be randomized into an intervention group and a control group based on a computer-generated randomization program with an allocation ratio of 1:1. The result of the group allocation cannot be masked to the patients and physicians who are responsible for delivering the intervention and follow-up. According to the study design, the patients and the physicians who take charge of

recruitment, supervision, and follow-up will not be blinded to the study groups. However, the physicians performing the assessments and data analysis will be blinded to the allocation status. Meanwhile, patients will be requested not to discuss their study group status with the physicians performing the assessments.

Data collection

The demographics and health information of the patients will be collected by the self-designed electronic questionnaire via the PFMT study app at baseline and will include age, height, weight, study centre, weight gain during pregnancy, menstruation, feeding patterns, occupation type and position, educational level, toileting position, smoking, family history, sexual activity, labour and physical activity and medical history. Obstetrical data, including the delivery date, gravidity and parity, delivery mode, newborn weight, will be collected from the electronic medical records. Both groups will be assessed by repeated questionnaires, physical examination and manometry at baseline (pretest) and 3 months (at the end-point of intervention). The validated questionnaires used to evaluate the outcomes will be completed by the patients via the PFMT study app each time before the assessment is performed. Physical examination and manometry will be performed by the same assessment physician at each participating centre who is blinded to the allocation status. The patients' adherence to and compliance with treatment will be subjectively recorded by a self-reported training dairy via the PFMT study app and objectively recorded by the biofeedback device in the intervention group. In the control group, the adherence and compliance will be simply recorded by a self-reported training dairy via the PFMT study app.

Intervention

We will ensure that all patients receive identical therapy, with the exception of the inclusion of pressure-mediated biofeedback. Each patient will be determined to be capable of contracting the PFMs correctly before leaving by being taught how to contract the PFMs through vaginal palpation and manometers at baseline. After randomization, both groups will be supervised in downloading the PFMT study app designed to guide patients through the study. The PFMT study app will provide a different operation interface and function according to the allocation status. Moreover, both groups will receive a one-page handout with the details of the training protocol, while the intervention group will receive a personal home-based biofeedback device (XFT-0010CK) and an operation video. The supervision physician will ensure that the intervention group can connect the vaginal biofeedback device to the PFMT study app using Bluetooth before leaving.

Both study groups will be instructed to follow the same training protocol during the first three months after randomization. The intervention group will be supervised in using the PFMT study app that is capable of providing visual feedback to the patient during the PFMT. The intervention group will also be asked to evaluate the PFM function via the device by themselves once every three weeks. The control group will receive the supervised PFMT based on the video provided in the PFMT study app. Both groups will be asked to upload the exercise diary via the PFMT study app according to the completion of the daily training. During intervention, the supervising physician will telephone each patient in both groups, asking questions about the performance of PFMT once every three weeks to provide motivation and guidance. If patients

experience any possible adverse events during the intervention, such as vaginitis due to the use of the intravaginal air-filled probe, they will be asked to report them at any time to the supervising physician, who will report to the study director to decide on a management plan. Training meetings will be conducted during the trial to ensure that all physicians are maintaining the protocols.

Training protocol

One reason why the results of previous studies were not convincing enough was that the PFMT program was not properly designed or the intensity of the PFMT regime was not sufficient. According to the 2019 NICE guideline on the management of UI and POP in women, PFMT programmes should comprise at least 8 contractions performed 3 times per day. An intensive training protocol was proposed by some studies, consisting of three sets of ten contractions at maximum intensity holding for 6 seconds each, three or four times a week.

Both study groups will be instructed to follow the same training protocol during the first three months after randomization. The protocol includes the performance of three sets of fast and slow contractions completed per day in the supine position. Each set lasts for 6 min and comprises three replicates. Each replicate consists of 8 maximal contractions (held for 6 seconds with a 6-second rest) followed by 4 fast contractions (held for 1 second with a 1-second rest) and a 30-second rest between each replicate.

Study device

The study device adopted in our study was designed and developed by the principal investigators of this study and Shenzhen XFT Medical Limited in September 2021 for scientific

research use only. The wearable PFMT study device named XFT-0010CK is a digital device with an intravaginal insert and a battery pack. The device has a screen that is able to show the real-time value of the vaginal resting pressure and voluntary contraction pressure in units of mmHg. The intravaginal insert is a vaginal air-filled probe with the gas filled automatically when it starts to work. The two parts are combined with a thin air tube. The whole device is connected to the PFMT study app by Bluetooth to record the progress of and compliance with PFMT. There are two modes of the device: assessment and training modes. In regard to the assessment mode, the device is able to assess the PFM strength by the patients themselves following the instructions. The training mode is the significant mode to guide patients to complete PFMT by following the training protocol and visualizing the real-time progress of PFM contraction via the PFMT study app. The biofeedback is provided according to the pressure collected by the vaginal air-filled probe during training.

APP

Patients in both groups will be instructed to download and install an app named the PFMT study app from the App Store, which is available for both Android and iOS smartphones and was developed by our research group in collaboration with software engineers based on the study protocol to guide both groups through the study as shown in Fig 1. In both groups, patients can complete the questionnaires via the app and receive a reminder within 1 week before the indicated time. Additionally, the app will send daily push reminders to both groups to remind them to complete daily PFMT. Both groups can upload the exercise diaries via the app. The app will also provide a different operation interface and function according to the

allocation status. The study device connects to the PFMT study app using Bluetooth. In the intervention group, the app can support the performance of the PFM contraction by providing visual biofeedback on the PFM contraction over time while storing the duration of, frequency of and compliance with training. The control group will use the same training video three times a day.

Outcome measures

Primary outcome measure

The primary outcome will be evaluated by the International Consultation on Incontinence Questionnaire-Urinary Incontinence short form (ICIQ-UI SF), a patient-reported outcome measure (PROM) for the severity of UI. The questionnaire will be administered in the Chinese and the Chinese version has been validated. It contains 4 items including urine leakage (score of 0 = never to 5 = always), amount of urine leakage (scores of 0, 2, 4, 6 for increasing amounts per episode), and impact of the urine leakage on life (score of 0 = not at all to 10 = a great deal) and types of leakage symptoms experienced in the previous 4 weeks. The ICIQ-UI SF score is the sum of the first three items ranging from 0 to 21 divided into mild (0–5), moderate (5–13) and severe (13–21). Higher scores reflect greater severity.

Secondary outcome measures

PFM strength

The PFM strength will be evaluated by subjective (vaginal palpation) and objective (manometer) measurements. During the assessments, each participant will be examined in the lithotomy position with an empty bladder and the head of the bed inclined at a 30° angle. The subjective

PFM strength will be measured by digital vaginal palpation based on the modified Oxford grading scale (MOS). Patients will be asked to perform a maximum contraction of the muscles to squeeze and lift against the resistance provided by the assessors' fingers in the vagina. The subjective PFM strength will be classified within the range of Grades 0-5, with higher grades reflecting better strength (0 (no contraction), 1 (flicker contraction), 2 (weak contraction), 3 (moderate contraction), 4 (good contraction), 5 (strong contraction)).

The objective PFM strength will be defined as the maximum voluntary contraction pressure (MVCP), which is referred to as the force-generating capacity of a muscle and evaluated through an intra-vaginal manometer (a vaginal balloon probe filled with 20 ml gas) connected to a PHENIX USB 8 neuromuscular stimulation therapy system, which is equal to a high-precision pressure transducer (Electronic Concept Lignon Innovation, Montpellier, France). MVCP will be calculated as the difference between the vaginal resting pressure (VRP) and its peak pressure during a maximum voluntary contraction. The patients will be asked to perform maximum contractions of the PFMs three times without using the abdominal, gluteal, or hip adductor muscles during the contractions. Each contraction will have an interval of 15 seconds. The outcome will be recorded as the average of three measurements. Higher pressure is related to greater strength.

Quality of life

Quality of life will be evaluated by the Chinese version of Incontinence Quality of Life Instrument (I-QOL) with 22 items. Its Chinese version has not been validated. The instrument has 3 subscales: (1) avoidance and limiting behaviour, (2) psychosocial impacts, and (3) social

embarrassment. All items are evaluated using a 5-point Likert-type scale. The scores will be summed and transformed to a 0 to 100 scale. Higher scores represent better quality of life.

Broome pelvic muscle self-efficacy scale (BPMSES)

The questionnaire employed in our study to evaluate the outcomes of self-efficacy is reliable and validated. We will use a Chinese version with a 23-item rating scale consisting of two domains: efficacy expectations and outcome expectations. In the efficacy expectations domain, participants will demonstrate how confident they are in performing PFMT. In the outcome expectations domain, participants will indicate their level of confidence that the training will prevent unwanted urine leakage. The score ranges from 0 to 100. The higher the score is, the greater the self-efficacy perceived by the participant.

Curing and improvement in SUI

Cure in SUI was defined as the total score on the ICIQ-UI SF=0 at the endpoint of 3 months of treatment. Improvement in SUI was defined as a reduction in the ICIQ-UI SF score of ≥3 points compared with the endpoint of 3 months of treatment at baseline.

Patient adherence to treatment

There is no universally accepted standard for adherence. In our study, patients' adherence to treatment will be determined as the frequency of exercises anticipated for 3 months of supervised treatment, which will be subjectively self-reported and objectively device-recorded. Patients' overall adherence will be categorized as follows: 50%≤= low adherence; 50%-75%= medium adherence; and ≥75%= high adherence.

Statistical analysis

The primary outcome of this study is the ICIQ-UI SF score. An ICIQ-UI SF minimal clinically important difference of 2.5 points was reported by Nyström E in 2015. We referred to two RCT studies reporting ICIQ-UI SF data as the primary outcome for women with SUI with 5-month and 6-month follow-up indicated an assumed standard deviation (SD) of 5. Another study determined a SD of 10 because they expected that the SD at the 24-month time point could possibly be as high as 10.According to our study design, it would be reasonable for us to expect a SD of 8.1 at the 12-month time point. On this basis, the sample-size calculation in our study is expected to be based on a significant difference of 2.5 points in ICIQ-UI SF scores (SD of 8.1) as the primary outcome between the groups. A sample size of 222 participants per group will detect this difference calculated through PASS 15, with a power of 0.9 and a two-sided significance level of 0.05. Thus, a total of 500 women will be recruited (250 women per group), allowing for approximately 10% loss to follow-up during the 12-month period.

The data will be analysed by SPSS version 26.0 (IBM, Armonk, New York, USA). Continuous variables conforming to a normal distribution are presented as the means ± standard deviations (SDs), within-group comparisons will be analyzed with paired-samples t tests, and two independent samples t tests will be used for between-group comparisons. If the data is nonnormally distributed and presented as medians and interquartile ranges, the Wilcoxon signed rank test will be used for analyses within the groups, and the Mann−Whitney U test will be used for comparisons between two groups. Categorical variables are presented as n (%), the chi-square test will be used for within-group comparisons, and Fisher's precision probability test will be used for between-group comparisons.The relationship between self-reported

adherence and device-reported adherence will be assessed using a scatter plot. $P < 0.05$ will be considered significant.

Data management

The data management system adopted in this study includes the following: a patient side for providing PFMT tasks to patients and collecting questionnaire results, side effects and PFMT data; a physician side for monitoring and managing the PFMT progress of patients; and a hospital side for managing the operation process and results of the physician side and patient side within each hospital.
