# Supplementary material for: Pressure-Mediated Biofeedback With Pelvic Floor Muscle Training for Urinary Incontinence: A Randomized Clinical Trial
Source: JAMA Netw Open. 2024 Nov 5;7(11):e2442925. doi: 10.1001/jamanetworkopen.2024.42925 (PMC11539013; doi:10.1001/jamanetworkopen.2024.42925)
Supplement: Supplement 2. — Data Sharing Statement [file jamanetwopen-e2442925-s002.pdf]

Data Sharing Statement

Wang. Pressure-Mediated Biofeedback With Pelvic Floor Muscle Training for Urinary Incontinence. *JAMA Netw Open*. Published November 05, 2024.
doi:10.1001/jamanetworkopen.2024.42925

Data
Additional Information: ClinicalTrials.gov Identifier: NCT05115864
Data available: Yes
Data types: Deidentified participant data
How to access data: Deidentified data is available upon request to Xiuqi Wang (mary9988@126.com)
When available: beginning date: 08-30-2026

Supporting Documents
Document types: None

Additional Information
Who can access the data: Researchers whose proposed use of the data has been approved
Types of analyses: For a specified purpose
Mechanisms of data availability: With a signed data access agreement
